# The complexity of titin splicing pattern in human adult skeletal muscles

**DOI:** 10.1186/s13395-018-0156-z

**Published:** 2018-03-29

**Authors:** Marco Savarese, Per Harald Jonson, Sanna Huovinen, Lars Paulin, Petri Auvinen, Bjarne Udd, Peter Hackman

**Affiliations:** 1 Folkhälsan Research Center, University of Helsinki, Helsinki, Finland; 2 Department of Pathology, Fimlab Laboratories, Tampere University Hospital, Tampere, Finland; 3 Institute of Biotechnology, University of Helsinki, Helsinki, Finland; 4 Vaasa Central Hospital, Vaasa, Finland; 5 Folkhälsan Institute of Genetics, Department of Medical Genetics, University of Helsinki, Biomedicum, Haartmaninkatu 8, Pb 63, 00014 Helsinki, Finland

**Keywords:** Titin, Titinopathies, RNA-sequencing, Exon usage, Alternative splicing events, Splicing pattern

## Abstract

**Background:**

Mutations in the titin gene (*TTN*) cause a large spectrum of diseases affecting skeletal and/or cardiac muscle. *TTN* includes 363 coding exons, a repeated region with a high degree of complexity, isoform-specific elements, and metatranscript-only exons thought to be expressed only during fetal development. Although three main classes of isoforms have been described so far, alternative splicing events (ASEs) in different tissues or in different developmental and physiological states have been reported.

**Methods:**

To achieve a comprehensive view of titin ASEs in adult human skeletal muscles, we performed a RNA-Sequencing experiment on 42 human biopsies collected from 12 anatomically different skeletal muscles of 11 individuals without any skeletal-muscle disorders.

**Results:**

We confirmed that the skeletal muscle N2A isoforms are highly prevalent, but we found an elevated number of alternative splicing events, some at a very high level. These include previously unknown exon skipping events and alternative 5′ and 3′ splice sites. Our data suggests the partial inclusion in the *TTN* transcript of some metatranscript-only exons and the partial exclusion of canonical N2A exons.

**Conclusions:**

This study provides an extensive picture of the complex *TTN* splicing pattern in human adult skeletal muscle, which is crucial for a proper clinical interpretation of *TTN* variants.

**Electronic supplementary material:**

The online version of this article (10.1186/s13395-018-0156-z) contains supplementary material, which is available to authorized users.

## Background

The *TTN* gene encodes titin, a muscle protein spanning from the Z-disk to the M-band within the sarcomere. The genomic structure of *TTN* is quite remarkable. It contains 364 exons (363 coding exons plus the first non-coding exon) and can theoretically generate more than one million splice variants [1, 2]. It also has a large repeated region with a high degree of complexity [1].

Titin isoforms have traditionally been classified in three main categories based on the presence of the N2A and N2B elements within the I-band region [3–5]. N2A isoforms (mainly expressed in the skeletal muscles) contain the N2A element, but not the cardiac-specific N2B element. On the contrary, N2B isoforms only include the cardiac-specific N2B element. N2BA isoforms, expressed in the heart, include both the N2B and N2A elements. N2A and N2BA isoforms also include additional exons, resulting in a higher number of Ig and PEVK domains in the I-band region.

Two further isoforms, named Novex-1 and Novex-2, are very similar to N2B but each of them includes an isoform-specific exon (exon 45 and exon 46, respectively). Finally, the Novex-3 isoform only contains the N-terminal part of titin due to an alternative stop codon in the Novex-3-specific exon 48.

Interestingly, specific exons included in the inferred complete metatranscript (NM_001267550.1) and referred to as metatranscript-only or meta-only exons are thought to be expressed only during embryonic development. Thereafter, they are not included in the canonical soleus-derived N2A skeletal muscle isoform, or in any of the five cardiac isoforms.

An extensive use of alternative splicing (AS) in different tissues or in different developmental and physiological states has been reported, resulting in a longer or smaller protein [2]. This reflects the global massive use of tissue-specific AS events (ASEs) which have been described in the skeletal muscle [6, 7].

Although the presence of multiple different transcripts originating from *TTN* gene as consequence of ASEs has been partly suggested by experimental evidence [1, 2, 8], we still lack a clear picture of the global exon usage and of the subsequent splicing profile of *TTN* muscular transcripts.

The introduction of RNA sequencing (RNA-Seq) methods has enabled a comprehensive study of the transcriptome [9]. Although early work focused on gene-expression analyses, RNA-Seq is a powerful tool for the identification and the study of alternative exon and splice site usage and of novel isoforms. It also allows an accurate quantification of relative transcript abundances [6, 10].

**Fig. 1:**
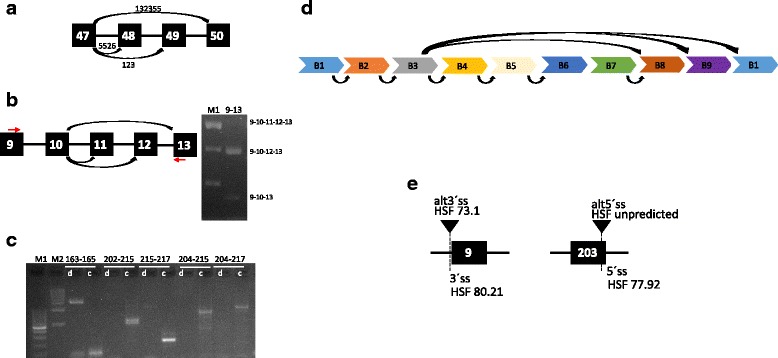
Isoform identification and titin alternative splicing events in human skeletal muscle. **a** The previously reported classes of isoforms differ from each other by the inclusion/exclusion of exons 48 (included only in Novex3 isoform) and 49 (included in all the other isoforms except the long skeletal muscle N2A-isoform). Our data suggests that N2A isoform is 20 times more expressed than Novex3. All the other isoforms have a very low expression. **b** We identified a low number of reads connecting exon 11 to its flanking exons. On the contrary, a high number of reads connect exon 10 to exon 12 and 13, thereby skipping exon 11. In line with the RNA-Seq results, a standard RT-PCR (forward primer on exon 9 and reverse primer on exon 13, red arrows) and agarose gel electrophoresis show a very low abundance of the transcript including exon 11. M1 = 100 bp ladder. **c** Several RT-PCRs and agarose gel electrophoresis show a variable expression of metatranscript-only exons, confirming the RNA-Seq results. In particular, no expression of exons 163 and 165 is detected; on the contrary, all the other RT-PCRs result in a detectable band corresponding to the expected size. M1 = 100 bp ladder; M2 = 1 kb ladder; d = PCR from a control DNA; c = RT-PCR from a control cDNA (obtained by a retrotranscription of RNA extracted from *gracilis* muscle). **d** Titin repeated region is composed of nine exons/blocks (here represented by different colors and named B1-B9) repeated three times. Within the repeated region, linear expression of consecutive exons has been detected. Moreover, a number of alternative splicing events has been identified. **e** We detected alternative splicing acceptors or donors leading to subtle changes in the produced protein. The splice-site strength for canonical splice sites (5′ss and 3′ss) as well as for alternative sites (alt 5′ss, alt 3′ss) has been calculated by Human Splice Finder (HSF)

In this study, we analyzed RNA sequencing data of human adult skeletal muscle tissues to obtain a comprehensive view of titin ASEs. This is crucial for a proper clinical interpretation of *TTN* variants that have been associated with a wide spectrum of human diseases and for an improved genotype-phenotype correlation [11–16].

## Methods

### Skeletal muscle samples and RNA extraction

Data was generated using 42 human skeletal muscle samples dissected from 12 anatomically different skeletal muscles (*tibialis anterior*, *flexor hallucis longus*, *soleus*, *extensor digitorum longus*, *gracilis*, *semitendinosus*, *semimembranosus*, *vastus medialis*, *vastus lateralis*, *sartorius*, *biceps femoris*, *adductor magnus*) collected from 11 adult individuals (7 males and 4 females) who had undergone above or below-the-knee amputation surgery for medical reasons other than neuromuscular disorders (Additional file 1:Table S1). A written informed consent was signed by all the patients and the Tampere University Hospital (Tampere, Finland) Ethics Committee approved the study.

The samples (5 × 5 mm of size) were processed immediately after their removal to avoid tissue degradation as previously described [17]. Total RNA was extracted from the selected samples by the TRIzol reagent method, according to the manufacturer’s instructions (Invitrogen, Life Technologies, Canada). RNA quality was checked with BioAnalyzer equipment using the RNA 6000 Nano Assay kit (Agilent Technologies, CA, USA).

### Library preparation, sequencing, and bioinformatics

Indexed sequencing libraries were generated from 1 μg of total RNA, using the TruSeq Stranded Total RNA kit according to the manufacturer’s instructions (Illumina, CA, USA). Single-end sequencing (86 bp reads) of multiplex libraries was performed on NextSeq500 instrument. Raw reads were mapped against the hg19 human reference genome using TopHat2 [18]. TopHat was also used for detecting and counting exon junctions. Alternative splice sites were evaluated using Human Splicing Finder (HSF) program [19].

For each exon, the inclusion rate was calculated as [(*I*/2)/[(*I*/2) + *E*], where *I* is the number of reads supporting the exon inclusion (all junctions going into and exiting the exon) and E is the number of reads supporting its exclusion.

### Experimental validation of alternative splicing events

For experimental validation of RNA-Seq results, cDNA synthesis was performed using SuperScript III First-Strand Synthesis System (Thermo Scientific, USA). RT-PCRs were performed using 1 μl of cDNA and a DreamTaq™ DNA Polymerase (Thermo Scientific). Primers were designed with Primer3 software (sequences available upon request). Amplified products were separated on 2% agarose gels and specific electrophoresis bands, corresponding to differently spliced products, were extracted using NucleoSpin Gel and PCR Clean-up (Macherey-Nagel, Germany) and analyzed by Sanger sequencing.

### Publicly available data

We also evaluated the presence of the ASEs in publicly available total mRNA sequencing data of adult *gastrocnemius medialis* from the ENCODE project (https://www.encodeproject.org; accession numbers ENCFF219LYV, ENCFF308RYZ, ENCFF569TCU, ENCFF408QZN, and ENCFF064NBB). Junctions were extracted from the available bam-files using regtools (https://github.com/griffithlab/regtools).

Similarly, we analyzed RNA-Seq data from fetal skeletal muscles (accession numbers ENCFF009MKH, ENCFF084FDS, ENCFF121PKV, and ENCFF405BHX), fetal heart (accession numbers ENCFF111DKK, ENCFF686KAP, ENCFF167WVS, and ENCFF174EGJ), and adult heart (accession numbers ENCFF735RZM, ENCFF834OIQ, ENCFF608FZD, and ENCFF621SXE) from ENCODE.

## Results

Before focusing on alternative splice events, we analyzed the canonical junctions, which are present in the previously reported isoforms, to evaluate their relative expression in human adult skeletal muscles. As expected, the junction 47–50, uniquely present in the previously identified skeletal long isoform N2A [1], is detected at very high level in all our samples (Fig. 1a). This confirms that most of the skeletal muscle transcripts belong to this class of isoforms.

We then calculated the number of reads supporting each of the N2A canonical splicing events (Additional file 2: Table S2). Most of the canonical N2A junctions were identified. Interestingly, we noticed a very low number of reads supporting the inclusion of exon 11 (junctions linking exon 10–11 and exon 11–12). Similarly, we did not observe reads connecting exons 183 and 203.

Then, we proceeded to the analysis of the alternative splice events. We identified 4039 unique splicing events, most of them in one or a few samples and supported by a very low number of reads. In order to eliminate the background sequencing noise and/or very weakly expressed transcripts, we applied a stringent quality control (QC)-filtering process, prioritizing only splicing events (*n* = 498) supported by at least 1000 reads and identified in at least 14 samples. To reduce possible artefacts due to technical issues and obtain a less biased splicing pattern, we analyzed publicly available total mRNA sequencing data of adult *gastrocnemius medialis* from the ENCODE project. In a very conservative approach, we only focused on splicing events identified in our experimental samples as well as in the publicly available data (> 10 reads in ENCODE data).

After that, we proceeded with a multistep analysis, based on two different categories of splicing events: (1) ASEs involving canonical splice sites (out of the repeated region and within this area) and (2) ASEs involving alternative splicing sites.

1. We identified 46 unreported exon junctions, involving canonical splice sites out of the repeated region (Table 1). All these 46 ASEs are predicted to maintain the frame. For 23 ASEs, we performed RT-PCR and all confirmed the RNA-Seq results.
  We identified three ASEs (10–12; 10–13; 10–14), suggesting the skipping of exon 11. In line with the RNA-Seq results, RT-PCR confirms that exon 11 was poorly expressed in human adult skeletal muscle (Fig. 1b).
  Interestingly, 24 unreported junctions span meta-only exons, suggesting their partial inclusion in *TTN* human adult skeletal muscle transcripts (Table 1 and Fig. 1c).
  We identified a high number of reads involving the canonical splice sites of exons included in the repeated area (Additional file 3: Supplementary Material 1, Additional file 4: Table S3 and Additional file 5: Table S4). Well-known bias due to such repetitive regions hampers a comprehensive and accurate study of this region. However, our data suggests the linear expression of consecutive exons within this area. We also identified a number of ASEs linking non-consecutive exons within the repeated elements (Fig. 1d).
2. We observed the usage of alternative splice sites (acceptors or donors) located next to the canonical sites. Most of these alternative splice sites (16/19) would produce an in-frame insertion or deletion of a few amino acids. The Human Splicing Finder (HSF) program displayed high splice site scores for most of these alternative splice sites, further suggesting their real use in *TTN* transcripts (Table 2 and Fig. 1e).

Based on the aforementioned splicing events passing our stringent QC filters, we calculated for each of the coding exons showing an alternative splicing, and not included in the repeated region, the number of reads supporting their inclusion or exclusion in *TTN* transcripts and a subsequent inclusion value (Table 3). It is noteworthy that 13 meta-only exons are expressed but only 7 have an inclusion value higher than 10%. On the other side, most of the canonical N2A exons, reported to be expressed in adult skeletal muscle, have a high inclusion value. Exon 11 as well as exons 155, 156, and 157 have an inclusion value lower than 50%, indicating that they are mostly spliced out.

**Fig. 2:**
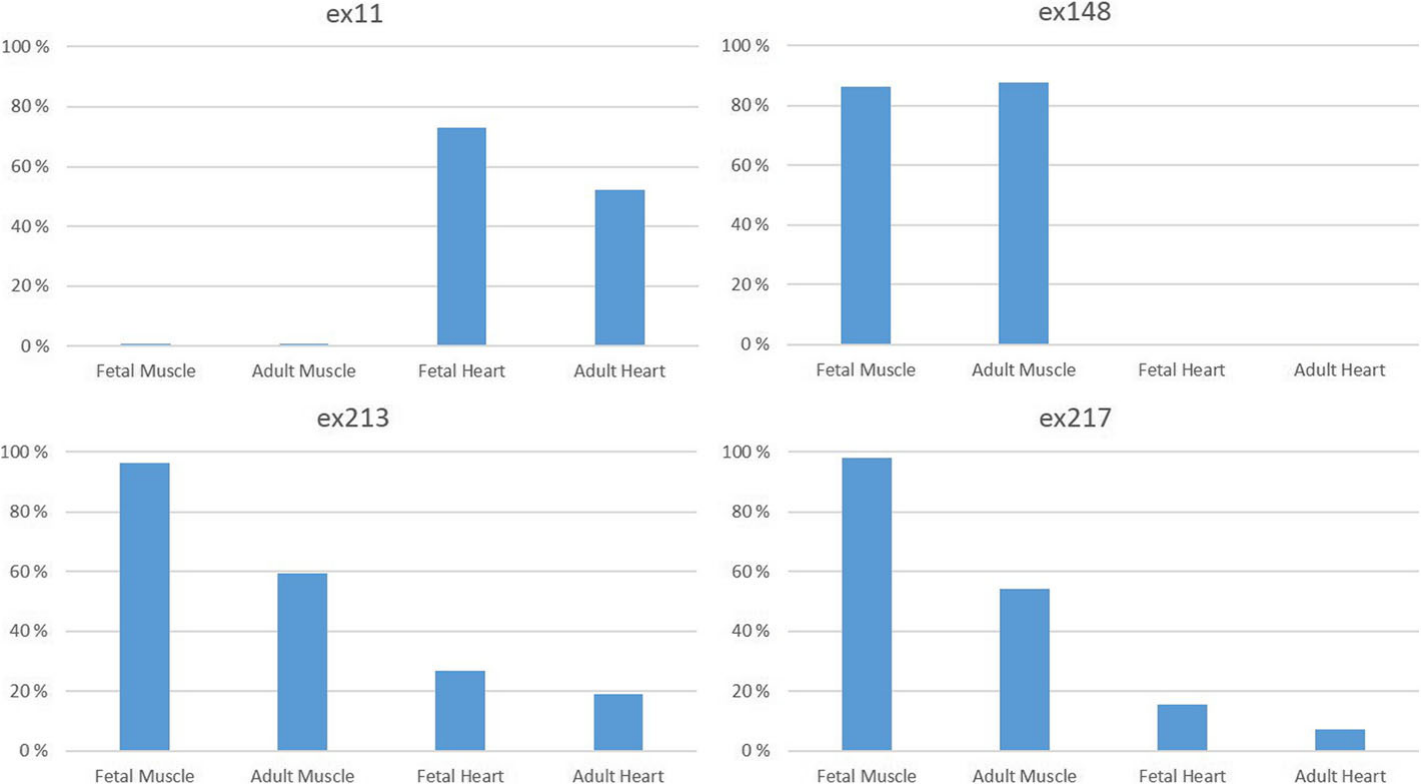
Comparison of alternative splicing events among different tissues at different developmental stages. The analysis of publicly available total mRNA sequencing data from the ENCODE project shows that exon 11 is expressed only in cardiac muscles, whereas the expression of exon 148 is limited to skeletal muscles. Exons 213 and 217 show an increased expression in fetal skeletal (and, at least in part, cardiac) muscle compared to the adult expression. The reported values correspond to the inclusion values, based on the number of reads supporting each exon inclusion or exclusion in *TTN* transcripts

To evaluate the spatial and temporal expression of exon 11 and of meta-only exons, we examined a subset of publicly available RNA-Seq data from fetal skeletal muscles and fetal and adult hearts (Fig. 2). Interestingly, exon 11 is mostly expressed in fetal and adult hearts. Its expression is very low in adult and fetal skeletal muscles. Exon 148 has a similar expression in fetal and adult muscles, and it is mostly skipped in fetal and adult hearts. On the contrary, meta-only exons 213–217 are almost constitutively expressed in fetal muscles and their expression is halved in adult muscles.

**Table 1:** Previously unreported junctions involving canonical splice sites out of the repeated region

Donor exon	Acceptor exon	#TotReads	#TotReads encode
10	12	63,681	31,420
10	13	37,864	22,425
10	14	25,860	19,371
36	38	2848	46
51	54	2319	52
85	88	8333	113
112	114	10,267	45
116	119	8651	1859
132	134	16,926	2843
137	143	1865	700
144	146	8300	297
146	151	10,262	805
146	152	3145	778
147	*148*	32,429	26,174
*148*	149	26,771	12,941
149	*150*	1304	951
*150*	151	1857	2836
153	158	12,300	116
154	158	35,902	4086
158	159	28,020	13,261
158	*167*	1129	45
158	*168*	3759	56
158	*171*	2965	279
158	172	7567	991
*159*	*167*	3117	2355
*159*	*168*	3192	1137
*159*	*171*	3722	3283
*159*	172	3627	1620
*167*	*168*	1830	2517
*168*	*169*	8111	7411
*169*	*170*	5910	5129
*170*	*171*	3237	8145
*171*	172	1439	8414
*202*	203	1358	15,787
203	209	1463	14
208	210	1741	27
212	*213*	28,311	11,949
*213*	*214*	8695	17,192
*214*	*215*	13,360	12,752
*215*	*216*	14,850	18,391
*215*	217	1647	1208
*216*	217	11,704	7775
*217*	218	10,983	15,909
219	222	1146	333
224	226	3126	2401
362	364	5458	2555

Metatranscript-only exons in italics

**Table 2:** List of events involving alternative splice sites

Donor	Acceptor	#Samples	#Reads	Frame	HSF consensus value novel donor splice site (value for wt)	HSF consensus value novel acceptor splice site (value for wt)	#Reads encode
c.669 (ex5)	c.673 (ex6-alt acc)	31	1195	Yes	–	78.86 (85.41)	287
c.1398 (ex8)	c.1399–3 (int8-alt acc)	42	6992	Yes	–	73.1 (80.21)	4456
c.9471 (ex40)	c.9508 (ex41-alt acc)	19	10,437	Yes	–	76.95 (90.97)	101
c.22528 (ex78)	c.22871 (ex80-alt acc)	27	1287	Yes	–	Unpredicted (77.00)	198
c.29124 (ex102-alt don)	c.29228 (ex103-alt acc)	31	3807	No	Unpredicted (88.47)	72.03 (79.27)	20
c.30754 (ex113)	c.30757 (ex114-alt acc)	16	1537	No	–	72.87 (85.71)	15
c.31426 (ex118)	c.31433 (ex119-alt acc)	31	4960	Yes	–	79.99 (81.96)	35
c.31762 (ex122)	c.31769 (ex123-alt acc)	19	3455	Yes	–	86.28 (67.88)	33
c.32197 (ex127)	c.32207 (ex128-alt acc)	25	1018	Yes	–	82.28 (78.21)	44
c.32392 (ex129)	c.32399 (ex130-alt acc)	41	5055	Yes	–	82.29 (75.58)	121
c.33910 (ex145)	c.33917 (ex146-alt acc)	18	1733	Yes	–	80.36 (77.55)	25
c.33994 (ex146)	c.34301 (ex148-alt acc)	19	12,820	Yes	–	72.21 (73.01)	159
c.38058 (ex191-alt don)	c.39484 (ex208-alt acc)	37	1038	Yes	Unpredicted (76.37)	74.68 (80.08)	32
c.38058 (ex191-alt don)	c.38980 (ex202-alt acc)	37	1427	Yes	Unpredicted (76.37)	75.98 (77.27)	26
c.39063 (ex203-alt don)	c.39484 (ex208-alt acc)	35	2300	Yes	Unpredicted (77.92)	74.68 (80.08)	29
c.39147 (ex204-alt don)	c.39484 (ex208-alt acc)	41	4744	Yes	Unpredicted (76.37)	74.68 (80.08)	105
c.40786 (ex223)	c.40790 (ex224 - alt acc)	30	1518	Yes	–	75.32 (94.42)	257
c.40876 (ex224)	c.40880 (ex225 - alt acc)	24	1635	Yes	–	77.2 (91.6)	151
c.44646 (ex243-alt don)	c.44914 (ex245)	20	10,706	No	83.39 (82.15)	–	15

*alt don* alternative donor, *alt acc* alternative acceptor

**Table 3:** Exon usage

Exon(s)	Inclusion rate	#Inclusion reads	#Exclusion reads	Skipping event
ex1-10	Constitutively expressed			
ex11	Constitutively spliced out			
ex12	54%	147,944	63,724	10–13;10–14
ex13	79%	194,924	25,860	10–14
ex14–36	Constitutively expressed			
ex37	98%	335,403	2848	36–38
ex38–44	Constitutively expressed			
ex45–46	Constitutively spliced out			
ex47	Constitutively expressed			
ex48	2%	5526	132,355	47–50
ex49	Constitutively spliced out			
ex50–51	Constitutively expressed			
ex52	98%	222,051	2319	51–54
ex53	98%	252,059		
ex54-ex78	Constitutively expressed			
ex79	99%	262,245	1287	c.22,528–22,871
ex80–85	Constitutively expressed			
ex86	91%	174,217	8333	85–88
ex87	93%	216,577		
ex88–112	Constitutively expressed			
ex113	90%	187,870	10,267	112–114
ex114-116	Constitutively expressed			
ex117	92%	200,426	8651	116–119
ex118	92%	209,844		
ex119-132	Constitutively expressed			
ex133	66%	66,307	16,926	132–134
ex134–137	Constitutively expressed			
ex138	97%	143,120	1865	137–143
ex139	96%	86,065		
ex140	95%	77,132		
ex141	96%	97,228		
ex142	96%	100,706		
ex143–144	Constitutively expressed			
ex145	83%	82,433	8300	144–146
ex146	Constitutively expressed			
ex147	62%	86,205	26,227	c.33994–34,301;146–151;146–152
*ex148*	68%	72,020	16,913	146–151;146–152;147–149
ex149	67%	53,414	13,407	146–151;146–152
*ex150*	4%	3161	35,240	146–151;146–152;149–151
ex151	94%	94,107	3145	146–152
ex152–153	Constitutively expressed			
ex154	82%	115,379	12,300	153–158
ex155	35%	52,812	48,202	153–158;154–158
ex156	34%	49,216		
ex157	40%	64,075		
ex158	Constitutively expressed			
*ex 159*	20%	52,844	103,121	158–167;158–168;158–171;158–172;158–173;158–175;158–182;158–184;158–191;158–193;158–204
*ex160-ex166*	Constitutively spliced out			
*ex 167*	2%	6076	123,699	158–168;158–171;158–172;158–173;158–175;158–182;158–184;158–191;158–193;158–204;159–168;159–171;159–172;159–173;159–175;159–184;159–193
*ex 168*	7%	16,892	116,748	158–171;158–172;158–173;158–175;158–182;158–184;158–191;158–193;158–204;159–168;159–171;159–172;159–173;159–175;159–184;159–193
*ex 169*	6%	14,021		
*ex 170*	4%	9147		
*ex 171*	5%	11,363	110,061	158–172;158–173;158–175;158–182;158–184;158–191;158–193;158–204;159–172;159–173;159–175;159–184;159–193
ex172-205	Repeated region			
ex206	81%	184,735	21,799	175–209;184–209;c.38058-c.39484;193–209;c.39063-c.39484;203–209;c.39147-c.39484
ex207	68%	91,407		
ex208	76%	86,726	13,717	175–209;184–209;193–209;203–209
ex209	97%	99,982	1741	208–210
ex210–212	Constitutively expressed			
*ex213*	26%	37,006	53,547	212–218
*ex214*	17%	22,055		
*ex215*	22%	29,857		
*ex216*	19%	26,554	55,194	212–218;215–217
*ex217*	19%	24,334	53,547	212–218
ex218-ex219	Constitutively expressed			
ex220	99%	168,189	1146	219–222
ex221	99%	194,654		
ex222-224	Constitutively expressed			
ex225	95%	124,798	3126	224–226
ex226-243	Constitutively expressed			
ex244	93%	285,487	10,706	c.44,646–44,914
ex245-362	Constitutively expressed			
ex363	91%	115,672	5458	362–364
ex364	Constitutively expressed			

Metatranscript-only exons in italics

A list of all detected ASEs that did not reach the minimum filtering criteria (i.e., a minimum of 14 out of 42 samples analyzed and at least 1000 supporting reads in total) or were not identified in the publicly available ENCODE data is included in Additional file 6: Table S5.

## Discussion

Recent mRNA-Seq transcriptomic analyses show that most of multi-exonic genes are alternatively spliced [7, 10, 20]. In particular, a vast majority of ASEs are tissue specific [10], and skeletal muscle seems to be among the tissues showing the highest numbers of tissue-specific ASEs [6, 7, 20].

Considering its 363 coding-exons and its genetic organization, a large number of ASEs were expected and partly reported in *TTN* transcripts. However, previous data, obtained by using different heterogeneous strategies in a pre-NGS era, did not provide a comprehensive view of the *TTN* splicing pattern and neither any unbiased repertoire of *TTN* ASEs in human adult skeletal muscles [1, 2, 8].

In our study, by performing RNA-Seq analysis using 42 adult human skeletal muscle samples, we identified in a reliable way a large number of ASEs, some of them at a very high level.

We detected previously undescribed exon-exon junctions, suggesting novel, unreported skipping events. Exon 11, included in the canonical adult skeletal muscle isoform N2A, is mostly skipped in adult skeletal muscles. On the contrary, most of the so-called metatranscript-only exons are expressed in adult skeletal muscle at a variable level. Moreover, we identified alternative acceptors and donors leading to subtle changes in the produced protein. Although these events need to be experimentally validated, similar ASEs have already been described in other human genes and their functional relevance has been hypothesized [21–23].

With the exception of exon 11, the N-terminal exons, coding for the Z-disk part of titin, are mostly constitutively expressed. Exons 8 to 14 encode for seven copies of a specific domain, named Z-repeat (Zr) [24]. In particular, exon 11 encodes for Z-repeat 4 [24], and its differential splicing has been previously reported [25]. Sorimachi and colleagues reported that Z-repeats 1, 2, 3, and 7 are expressed in all striated rabbit muscles, whereas the expression of Zr4, 5, 6 (corresponding to exons 11–12 and 13) is dependent on developmental stage and tissue-type [25]. The differential splicing of the titin Z-disk seems to be part of a larger and more complex process able to modulate Z-disk interactions via splicing regulation. The N-terminal Z-disk region of titin binds a number of proteins, including alpha-actinin, nebulin, and filamin C that undergo a similar process of differential splicing [26–28].

As expected, most of the ASEs occur in the I-band region of titin, where a large number of exons are alternatively spliced [3, 4]. It is noteworthy that exon 148, thought to be a meta-only exon, has an inclusion rate comparable to that of its neighboring exons in both adult and fetal skeletal muscles. Moreover, our experimental data as well as publicly available data suggests a significant expression of the meta-only exons 213, 214, 215, 216, and 217 in adult skeletal muscle, although their inclusion is higher in fetal muscles. In the M-band, we identified the previously reported splicing event (skipping of exon 363), producing the so called is7– and is7+ isoforms [29, 30]. In line with previous data, exon 363 is skipped in about 10% of *TTN* transcripts in human adult skeletal muscle.

As already discussed for the Z-disk splicing events, the regulation of alternative splicing events probably corresponds to modulation of interaction networks. For example, it is well known that the alternatively spliced is7 region, encoded by exon 363, binds the calcium-dependent protease calpain 3 (CAPN3) [31]. On the other hand, the role of the titin, and also nebulin, filament length (as a result of splicing events) on the sarcomere length and its passive elastic properties is still under debate [32–34].

Mutations in the *TTN* gene cause several different and heterogeneous skeletal muscle disorders with or without cardiac involvement, characterized by a variability in the age of onset, muscle involvement, and disease-course [11, 12, 35]. In addition, truncating mutations (*TTN*tv) have been associated with dilated cardiomyopathy (DCM) [13, 14]. A genotype–phenotype correlation has been observed to some extent [11, 15]. Mutations in metatranscript-only exons have recently been associated with a congenital titinopathy, characterized by arthrogryposis multiplex congenita and severe axial hypotonia as a form of congenital amyoplasia without cardiac involvement [36]. The hypothesis is that metatranscript-only mutations (mostly truncating mutations) specifically and selectively affect developmental isoforms, leading to a prenatal or congenital phenotype with a stable postnatal disease-course or weakness amelioration. On the contrary, proximal truncating mutations in canonical exons expressed on both alleles in adult isoforms lead to a premature truncated protein with nonsense mediated decay and would probably cause fetal death. The pathogenesis of *TTN*tv-related cardiomyopathies is probably more unclear; their penetrance is markedly reduced and they show a positional effect [14]. In particular, only *TTN*tv occurring in constitutive exons are significantly associated with DCM [14].

Deciphering the effective expression pattern of each *TTN*-exon, including meta-only exons, is crucial for a better understanding of *TTN*-related disorders. Our data clearly shows a variable expression for most of the meta-only exons (148, 150, 159, 167–171, 213–217), confirming, however, that some of them (160–166) are not expressed at all in human adult skeletal muscles. Our findings suggest the need for a more careful interpretation of the variants identified in a clinical setting.

Here, we provided an accurate inventory of ASEs in human adult skeletal muscles, which suggest the presence of a high number of undescribed isoforms. Moreover, taking into account all the alternative splicing events occurring in *TTN*, we calculated a reliable inclusion value for titin exons.

Further work remains to be done in order to refine our results. Long-read sequencing technologies, for example, will allow the identification of multiple splicing events along the same molecule, thereby elucidating how the individual splice events here described are connected, and thus confirming the presence of unreported isoforms. Similarly, a larger number of samples from each skeletal muscle type has to be analyzed in order to identify muscle-type specific ASEs or splicing patterns, considering that the current experimental setting has not identified any clear splicing difference among the muscles analyzed (Additional file 7: Table S6).

The exonic usage and the subsequent isoform expression seem to be finely regulated among different developmental and physiological and/or pathological states [2, 17, 37]. A further refinement of *TTN* expression profiling in different tissues and/or different physiological and pathological states (including regenerating or injured muscles) would be of a great clinical relevance, deepening, for example, our understanding of the role of *TTN* variants in complex human diseases.

## Conclusions

We have identified and partly characterized a large number of alternative splicing events in titin, providing the first RNA-Seq-based, accurate and comprehensive picture of *TTN* splicing pattern in adult human skeletal muscle. This same approach will probably unveil similar complex splicing patterns for other muscle transcripts.

## Additional files

Additional file 1: **Table S1.** List of samples analyzed. (XLSX 11 kb)

Additional file 2: **Table S2.** N2A splicing junctions. (XLSX 44 kb)

Additional file 3: Supplementary Material 1: Titin repeated region. (DOCX 112 kb)

Additional file 4: **Table S3.** Previously reported junctions in the repeated region. (XLSX 11 kb)

Additional file 5: **Table S4.** Unreported junctions involving exons in the repeated region. (XLSX 12 kb)

Additional file 6: **Table S5**. List of alternative splicing events not reaching the minimum filtering criteria or not identified in the publicly available ENCODE data. (XLSX 155 kb)

Additional file 7: **Table S6.** Previously unreported junctions clustered accordingly to specific skeletal muscle types. (XLSX 17 kb)

## Abbreviations

AS: Alternative splicing
ASEs: Alternative splicing events
HSF: Human Splicing Finder
NGS: Next-generation sequencing
PCR: Polymerase chain reaction
QC: Quality control
RNA-Seq: RNA sequencing
RT-PCR: Reverse transcriptase-polymerase chain reaction
*TTN*tv: Titin truncating variants
