# Supplementary material for: The complexity of titin splicing pattern in human adult skeletal muscles
Source: Skelet Muscle. 2018 Mar 29;8:11. doi: 10.1186/s13395-018-0156-z (PMC5874998; doi:10.1186/s13395-018-0156-z)
Supplement: Supplementary file 3 — Supplementary Material 1: Titin repeated region. (DOCX 112 kb) [file 13395_2018_156_MOESM3_ESM.docx]

**Supplementary Material 1 – Titin repeated region**


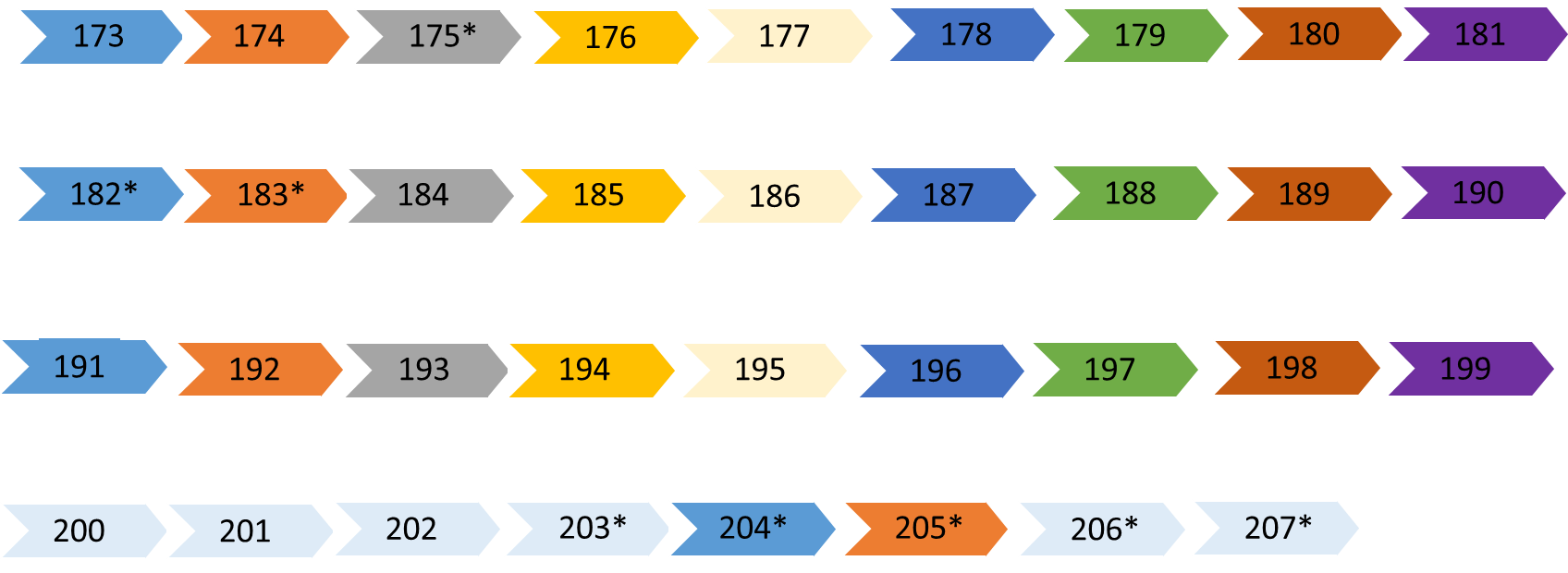


Titin repeated region is composed of nine exons (here represented by different colors) repeated three times. Therefore, by using an NGS traditional approach, each ´copy´ of a repeated exon is indistinguishable from the others (for example, exon 173 is indistinguishable from exons 182 and 191; 174 is indistinguishable from exons 183 and 192 and so on). Moreover, two further exons (204 and 205) are a copy of previous exons.

Within the repeated region, the only expressed exons in human adult muscle are 175-182-183-203-204-205 (indicated by an asterisk). The remaining exons are thought to be metatranscript-only exons.

During the mapping phase, NGS-reads are split among the repeated exons, i.e. reads spanning exons 173 and 174 are mostly indistinguishable from those spanning exons 182-183; 191-192; 204-205; 173-183; 173-192; 173-205; 182-192; 182-205 and 191-205. In other words, it is possible to detect reads linking ‘the blue exons’ (173-182-191-204) to the ´orange ones´ (174-183-192-205) without reliable information about the specific exons connected.

In order to overcome the technical issues due to such a complex genetic organization, we used a pragmatic approach.

1) We counted all reads supporting expected, canonical junctions (Additional file 4 – Table S3);

2) We counted all the reads suggesting previously unreported linear junctions (Additional file 5 – Table S4);

3) We counted all the reads supporting not canonical junctions, i.e. connecting exons out of the repeated area and exons within the repeated region (Additional file 5 – Table S4);

4) We counted all the reads supporting not canonical junctions, i.e. connecting exons within the repeated region (Additional file 5 – Table S4).

Similarly, we included in Table S3 and S4 the number of reads supporting each junction identified in publicly available Encode data from adult skeletal muscle (*gastrocnemius medialis*).
